# Rainfall-driven *E. coli* transfer to the stream-conduit network observed through increasing spatial scales in mixed land-use paddy farming karst terrain

**DOI:** 10.1016/j.wroa.2019.100038

**Published:** 2019-10-10

**Authors:** Sarah J. Buckerfield, Richard S. Quilliam, Susan Waldron, Larissa A. Naylor, Siliang Li, David M. Oliver

**Affiliations:** a Biological and Environmental Sciences, Faculty of Natural Sciences, University of Stirling, Stirling, FK9 4LA, United Kingdom; b School of Geographical and Earth Sciences, University of Glasgow, Glasgow, G12 8QQ, United Kingdom; c Institute of Surface-Earth System Science, Tianjin University, Tianjin, 300072, China

**Keywords:** Catchment hydrology, Faecal indicator organism, Faecal pollution, Storm event, Water quality

## Abstract

Karst aquifers have distinctive hydrology and supply 25% of the world’s population with drinking water, making them a critical geological setting for understanding and managing microbial water pollution. Rainfall causes elevated concentrations and loading of faecal microorganisms, e.g. *E. coli*, in catchment surface and groundwater systems, increasing the risk of human exposure to faecally-contaminated water. However, effective management of microbial water quality in complex karst catchments is constrained by limited understanding of *E. coli* - discharge responses to rainfall. We analysed how rainfall events of varying magnitude (2.4–100 mm) control *E. coli-*discharge dynamics at increasing spatial scales in a mixed land-use karst catchment in southwest China. During the wet season, hourly water sampling was undertaken throughout five storm events to characterise in high detail *E. coli* emergence with resulting flow across multiple sites of varying catchment area, stream order, and land-use. *E. coli* concentration was found to increase by 1–3 orders of magnitude following rainfall events. Maximum *E. coli* concentration and speed of *E. coli* recession were influenced by rainfall (amount, intensity), timing of agricultural activities, and position in the hydrological system. For high intensity events ∼90% of the cumulative *E. coli* export occurred within 48 h. *E. coli* concentration increased with increasing discharge at all sites. *E. coli* concentration at low discharge was higher in the headwaters than at the catchment outlet, while the rate of increase in *E. coli* concentration with increasing discharge appears to follow the opposite trend, being higher at the catchment outlet than the headwaters. This was attributed to the decreasing flow path gradient and increasing degree of development of the fissure network, but further event monitoring at varying catchment scales is required to confirm this relationship. The results provide novel insight into how rainfall characteristics combine with land-use and catchment hydrology to control *E. coli* export in karst landscapes.

## Highlights

• Rainfall, land-use & catchment hydrology control *E. coli* export in karst landscapes.
• *E. coli* concentration increased by 1–3 orders of magnitude following rainfall events.
• Under high intensity rainfall ∼90% of cumulative *E. coli* export occurred within 48 h of the event.
• Both urban and agricultural land can contribute high *E. coli* load to receiving waters.

## Graphical abstract

**Figure:**
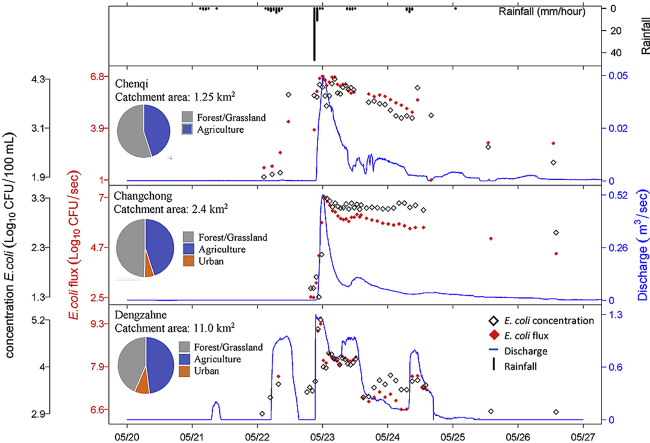

## 1 Introduction

Karst aquifers provide 25% of the global population with drinking water (Hartmann et al., 2014). Understanding and managing microbial water pollution in karst environments is therefore key for protecting the health of those reliant on this source of drinking water. Karst catchments respond differently to rainfall than granular aquifers due to the extreme anisotropy in hydrological properties such as hydraulic conductivity and transmissivity, and direct connectivity between the surface and groundwater systems (Bakalowicz, 2005; Fu et al., 2016). Effective hydraulic conductivity can vary by 10–12 orders of magnitude within a karst aquifer, due to the high velocity pathways provided by dissolution-developed conduits and the very low porosity of the primary rock matrix (White, 2018). Following rainfall, contaminants carried in runoff can bypass the soil profile and be flushed directly into karst aquifers through sinkholes and depressions. Faecal microbes stored in the fracture matrix or deposited in stream bed sediment during low flow conditions can survive for long periods, and be remobilised during storm events (Padilla and Vesper, 2018; Pronk et al., 2007). Hydrological transfer via the karst conduit structure to drinking water bores and springs thus presents a significant pathway for human exposure to microbial pollutants such as *E. coli*, the most commonly-used indicator microorganism for inferring faecal contamination (Epting et al., 2018).

Rainfall-runoff processes can facilitate the mobilisation, transfer and delivery of pollutants from land to receiving waters (VanWormer et al., 2016). In-situ monitoring capability for nutrients has demonstrated how high resolution datasets are invaluable in understanding how rainfall variables interact with karst hydrology and source availability (Yue et al., 2019). However, microbial water quality response to different combinations of faecal inputs and hydrological drivers (such as rainfall) is poorly understood, particularly in karst terrain (Vermeulen et al., 2015). This is partly because, unlike with nutrient pollution, in-situ high-resolution sampling combined with on-site analysis has not yet translated into a standard operating procedure for the quantification of *E. coli*, other faecal indicator organisms (FIOs), or human pathogens. Monitoring of FIO-discharge (Q) relationships through storm events is therefore challenging. It is further compounded by regulatory requirements to monitor microbial pollution at end-point receptors and locations of likely human exposure risk, such as bathing or shellfish harvesting waters, rather than quantifying FIOs distributed across catchment drainage networks (Oliver et al., 2016).

Catchments can comprise a mosaic of land-use types containing a suite of diffuse and point sources of FIOs. Diffuse FIO pollution dominates in rural catchments, with leaking septic tanks and slurry pits representing largely unquantified FIO loading (Sowah et al., 2017). In SE Asia, paddy farming dominates large areas of karst land. The health impact of using wastewater to irrigate paddy crops is starting to receive research attention (e.g. Barna, 2019) but, relative to grassland research, data are scarce (Buckerfield et al., 2019). In urban areas, failure and flooding of point sources, e.g. sewage treatment plants, are the leading sources of microbial contamination during rainfall events (Sauer et al., 2011). However, hydrological connectivity controls the delivery of FIOs from land to water and even in non-karst catchments the role of hydrological connectivity in driving the dynamics of FIOs in streams remains poorly characterised (Neill et al., 2018).

Despite their complexity, catchments remain a fundamental unit for framing water management decisions (Cho et al., 2016). However, those management decisions are constrained by data availability and limited modelling of FIO flux from catchments under low and high flow conditions (Vermeulen et al., 2015). Such modelling also requires better appreciation of responses in microbial water quality across different spatial and temporal scales (Muirhead and Meenken, 2018). Some studies have quantified FIO emergence with storm hydrographs (e.g. Murphy et al., 2015; Oliver et al., 2015; Ridley et al., 2014), but how rainfall events of varying magnitude, and successive rainfall events, impact on FIO – Q dynamics at different spatial scales within catchments is poorly documented (Buckerfield et al., 2019). Comparable research in karst terrain is scarcer yet, and requires investigation due to the unique hydrology and risk factors that can influence drinking water contamination. This form of dataset is required to inform on the risk presented by different sources under varying rainfall conditions, and it will provide the evidence base to inform where mitigation is best targeted.

The aim of this study therefore, was to investigate the impact of rainfall events of varying magnitude on *E. coli* – Q dynamics at increasing spatial scales in a typical mixed land-use karst catchment. Specifically, the objectives of our multi-site, multi-scale approach were to: (i) characterise *E. coli* – Q dynamics following rainfall, and determine the influence of different rainfall characteristics and antecedent conditions, and timing of agricultural activities on this relationship; and (ii) assess how rainfall-driven concentration and load of *E. coli* in receiving waters varies across land-use composition, variation in hydrological structure, and increasing scale of the contributing catchment area.

## 2 Materials and methods

### 2.1 Study catchment

**Fig. 1:**
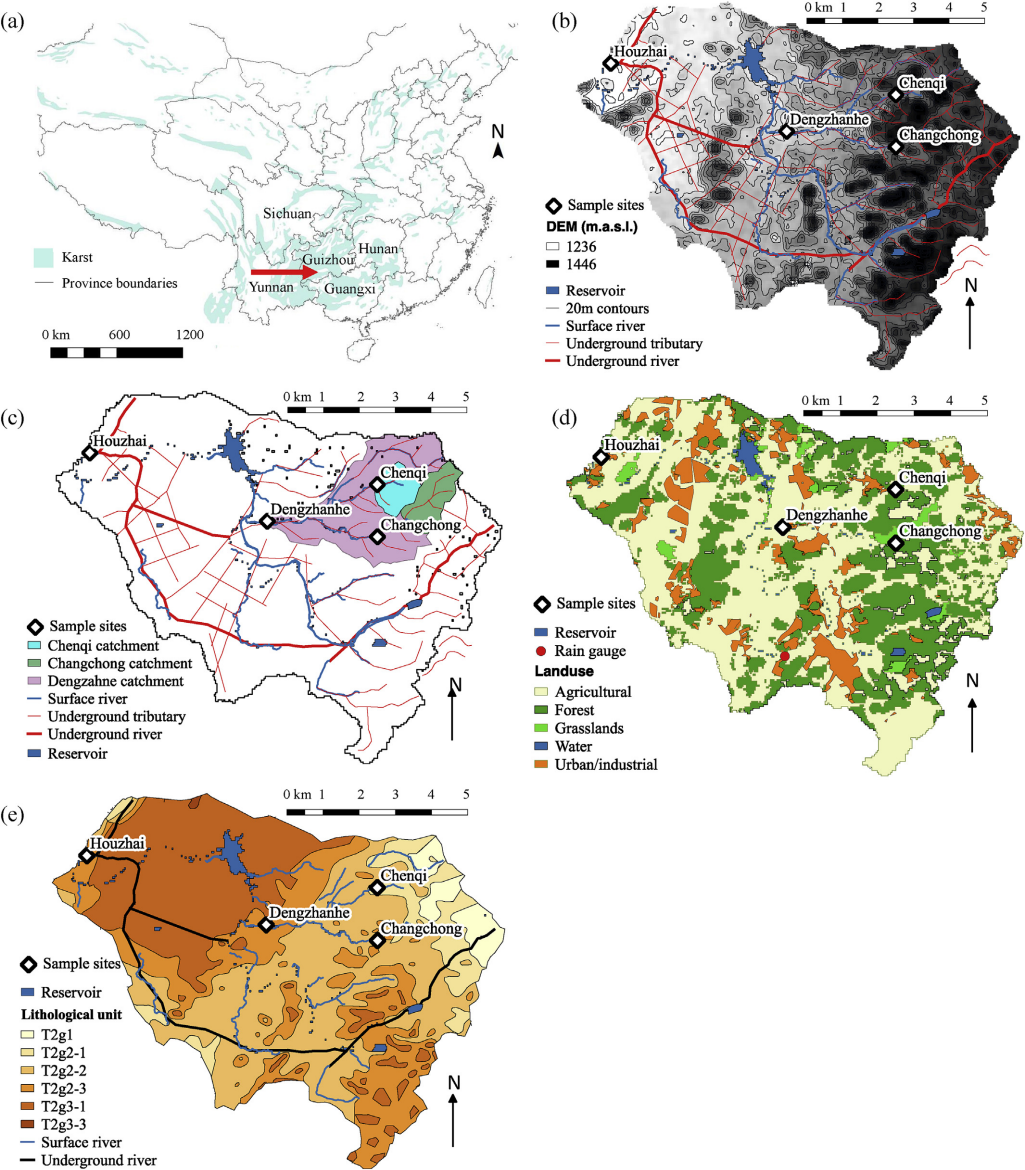
**(a)** Distribution of exposed karst in China, red arrow shows location of study catchment. Map layers sourced from Harvard WorldMap, Karst areas of China and Provinces of China (1997). **(b)** Sampling sites shown on a digital elevation model of HZ catchment with surface streams, the major underground conduit, and minor underground tributaries shown. **(c)** Watersheds for the four sampling sites (Chenqi (CQI), Changchong (CC), and Dengzahne (DZ) are shaded, and Houzhai (HZ) is the entire catchment). DEM, land-use, and geological maps courtesy of Puding karst ecosystem research station, Guizhou Province, China. **(d)** Land-use in the HZ catchment. Urban and industrial land was updated based on digitisation from Google Maps, 2016), accessed through QGIS v.2.14.10 open layers plugin and field validated in May-June 2017. **(e)** Geology of HZ catchment. T^2^g^1^ is interbedded shale and marlstone, T^2^G^2−1^ is limestone with vermicular limestone, T^2^g^2−2^ is limestone with interbedded marlstone, T^2^g^2−3^ is limestone, and T^2^g^3−1^ and T^2^g^3−3^ are dolomite. The ratio of CaO:MgO in these strata is estimated to be 48,28,30, and 3 for T^2^G^2−1^ T^2^g^2−2^ T^2^g^2−3^ T^2^g^3^ respectively. (For interpretation of the references to colour in this figure legend, the reader is referred to the Web version of this article.)

The Houzhai (HZ) catchment drains a land area of 73.5 km^2^ and is located at the centre of the southwest China karst region (Fig. 1 a), which is one of the most extensive karst regions in the world. This region has the highest national poverty rates and experiences high intensity rainfall events during the wet season (Cao et al., 2015). A number of sub-catchments in the HZ catchment were instrumented with in-situ hydrochemistry probes and pressure transducers for monitoring water levels. The locations of the four sites used in this study are shown in Fig. 1. Sites were selected to represent examples of contrasting land use, increasing contributing catchment area and to capitalise on existing monitoring infrastructure. The topography of the catchment ranges from mountainous karst cone-depression landforms in the east (maximum elevation 1565 m) to dominantly flat plains in the west (minimum elevation 1218 m) (Fig. 1 b). The headwater sub-catchments are characterised by steep topography and thin soils (<50 cm), and fast infiltration and vertical recharge of groundwater through sink holes and shafts, resulting in rapid responses in discharge and return to base-flow (Zhang et al., 2017). Further downstream the topographic and hydraulic gradients diminish, soil cover becomes thicker, and the fissure network becomes well developed, resulting in attenuation and storage of recharge (Liu et al., 2010). One well-developed continuous conduit running along the southerly boundary (Fig. 1 c) acts as the primary drainage of the underground water system, and a fissure-controlled conduit network in the central and western area of the catchment is well connected with the primary conduit (Zhang et al., 2017). The drainage system for the northerly sub-catchments (including Chenqi (CQI), Changchong (CC), Dengzahne (DZ), and Qingshan (QS) reservoir) (Fig. 1 c) is a modified series of transitions between the surface and groundwater systems. The headwater catchments CQI and CC drain primarily through springs, which are artificially channelled into concrete surface water channels that discharge into Qingshan reservoir. Reservoir outflow continues as a surface river to the outlet of the HZ catchment.

The catchment is a typical mixed-land-use paddy farming region with approximately half of the land used for cropping (major crops being rice, rapeseed, and maize, with additional crops including soybeans, vegetable, and fruit) (Fig. 1 d). CQI is an entirely agricultural headwater catchment, with dominant crops being rice, rapeseed, corn, and soybeans. CC contains paddy fields and urban land in the upper reaches of the catchment (∼3 km from the outlet), with land closer to the outlet accommodating dry-land cropping similar to CQI but also significant fruit crops and forested hillslopes. DZ catchment contains a higher percentage of urban land (Table 1) including land directly adjacent to the sampling location. The HZ groundwater outlet is located within a village at the outlet of HZ watershed. The primary southern conduit discharging at HZ runs through agricultural land and several villages. Connectivity between the outlets of Dengzhanhe and HZ catchments is highly fragmented, with possible hydrological flow paths through the QS reservoir and the conduit system. The majority of steep hillsides are forested, and much of the remaining flat land is urban, distributed as small towns and villages (population 53,500 - statistics from Puding county government). The lithology comprises five stratigraphic units of the Middle Triassic Guanling Formation, generally decreasing in age from east to west (Figure e).

**Table 1:** Summary characteristics of sample site locations. Land-use areas were derived from interpretation of google satellite imagery and GIS datasets of land-use (Google Maps, 2016. Accessed through QGIS v.2.14.10 open layers plugin. GIS datasets courtesy of Puding karst ecosystem research station).

Site	Classification	Drainage area (km^2^)	Land-use (%)
CQI	Headwater catchment underground outlet	1.25	Agriculture: 45 Forest: 50 Grassland: 5
CC	Headwater catchment underground outlet	2.4	Urban: 5.4 Agriculture: 45 Forest: 40 Grass: 9.4
DZ	Surface water monitoring point, outlet of sub-catchment including CQI and CC tributaries	11.0	Urban: 8.5 Agriculture: 48 Forest: 37 Grassland: 5
HZ	HZ watershed highest order spring outlet	73.5	Urban: 12.1 Agriculture: 51 Forest: 33 Grass:3

### 2.2 *E. coli*, discharge, and turbidity data collection

#### 2.2.1 In situ hydrological monitoring

Weirs at the designated monitoring points provided infrastructure for continuous Q measurements and sampling of water quality parameters. Each gauging station was equipped with a water level logger (GB/T3091-2008 pressure transducer) and an Aqua TROLL 600 multiparameter sonde, which continuously logged in-stream turbidity and temperature. Pressure transducers were built into a stilling well and provided water depth measurements at a 5-min interval for later conversion to stream Q using an established rating curve for each site (Zhang et al., 2017). Rainfall data was obtained from a rain gauge at Lahoetain in the southern region of the catchment (Fig. 1). Standard aseptic grab sampling was not possible due to the remoteness of sites and therefore automatic water samplers (Qingdao SuYuan Environmental protection equipment Co. Ltd, China) were used for capture of storm-related water samples. Deploying autosamplers in this study was further justified on the basis that: (i) the *E. coli*-Q ‘patterns’ were being compared; and (ii) relative rather than absolute differences between *E. coli* values were considered important for comparing across sites given that concentrations were not being assessed against regulatory standards. The auto-samplers were housed within concrete infrastructure adjacent to the weirs, which helped to keep the samples cooler than ambient temperature, reducing potential for temperature induced *E. coli* die-off before analysis (Pope et al., 2003). All samples were retrieved, returned to the laboratory in a cool-box, and analysed within 20 h of sampling.

#### 2.2.2 Storm monitoring

To determine the concentration of in-stream *E. coli* in response to rainfall and resulting Q, five rainfall events were sampled at one or more of the four monitoring sites, over a two-month period from the beginning of the wet season. For the purposes of this study, rainfall events are defined by the amount and intensity of rainfall, as this is constant across all sites while the discharge response varies. To minimise contamination, auto-sampler bottles were triple-sterilised with ultra-pure boiling water (autoclaving was not possible), and sealed with aluminium foil until deployed in the field prior to all storm events, as per Oliver et al. (2015). Sample collection began a minimum of 2 h prior to the commencement of rainfall. Samples were collected at an hourly interval for 24 h, then at two-hourly intervals from 24 to 48 h, and at diminishing frequency (typically every 6 h) from 48 to 96 h. Simultaneous sampling at three monitoring sites was carried out over two events at (i) CQI, CC, and DZ, and (ii) CQI, DZ, and HZ. An additional two events were monitored at CQI only, and one further event at HZ only.

#### 2.2.3 Microbiological analysis

*E. coli* were enumerated using the standard UK Environment Agency method of membrane filtration (EA, 2009). Each water sample was vacuum-filtered through a sterile 0.45 μm cellulose acetate membrane (Sartorius Stedim Biotech., Goettingen, Germany) and analysed in duplicate using a sterilised filtration unit (Rocker Scientific Co, Taiwan). The membrane was then aseptically transferred to a plate containing Membrane Lactose Glucuronide Agar (MLGA) (CM1031, Oxoid, Basingstoke, UK) and incubated at 37^o^C for 24 h for the determination of presumptive *E. coli* colonies. Sample volumes ranging between 1 and 100 mL were filtered to capture between 20 and 200 *E. coli* colony forming units (CFU), with further 1:10 serial dilutions in phosphate buffered saline (PBS) filtered where appropriate. Method blanks were regularly used to assess aseptic technique and to evaluate sterilisation efficiency between samples. All data are reported as CFU 100 mL^−1^.

### 2.3 Data analysis

All statistical analysis and modelling were performed using R v.3.6.0 (R Core Team, 2019). All *E. coli* counts underwent log_10_ transformation prior to statistical analysis. Linear regression modelling of the *E. coli* – Q and *E. coli* – turbidity relationships was performed, investigating different combinations of ‘Site’ and ‘Event’ as categorical predictors interacting with discharge and turbidity as the continuous predictors. The goodness of fit of models was ranked using Akaike’s Information Criterion (AIC) values, with a threshold *delta* AIC value of 7 used to select candidate models (Fabozzi et al., 2014). Due to turbidity data only being available for two sites, and only one shared event at both sites, separate models were developed for the two sites (CQI and HZ). The *F*-statistic was used as a measure of the significance of candidate models and the significance of the effect of predictors on the response variable was assessed using *t*-values (both at p < 0.05 significance level). Adherence to the assumptions of regression was checked by inspection of the normality of residuals and model diagnostics. Relationships between maximum Q, *E. coli* concentration and flux, event export, and rainfall characteristics were also assessed following the same methodology. Analysis of *E. coli* – Q hysteresis was undertaken on those events where a hydrological response to rainfall resulted in a well-defined Q peak comprised of a rising and falling limb. All storm event *E. coli*-Q responses were examined visually for the presence and direction of hysteretic loops. Discharge and *E. coli* concentration data were linearly-interpolated and discretised into units of seconds. Total event export was calculated by linear interpolation and trapezoidal integration of the derived *E. coli* flux at 1 s intervals (McKergow and Davies-Colley, 2010). The duration of the event was taken as the duration of monitoring.

## 3 Results

The events monitored during the study varied spatially with respect to their rainfall-runoff signatures and associated FIO export (Table 2). An explanation of how each parameter was derived is given in supplementary information (Table 5). Events are referred to by their assigned number. All blanks yielded zero colony forming units. The average water temperature was lowest in the headwater catchments CQI and CC (16.6°C), 18.3 at HZ°C, and 19.8°C at DZ (supplementary information, Table 6).

**Table 2:** Summary table of key parameters describing each Event. Table 5 in the Supplementary Information gives details of how each parameter was calculated.

Site (Event #)	Date	Maximum *E.coli* concentration (CFU 100 mL^−1^)	Maximum discharge (m^3^ s^−1^)	Maximum *E.coli* flux (CFU s^−1^)	Total *E.coli* export (CFU)	Rainfall amount (mm)	Maximum rainfall intensity (mm h^−1^)	Event loading (CFU km^−2^)
CQI (1)	24/04/2017	1.2 × 10 ^3^	9.6 × 10 ^−4^	2.3 × 10 ^3^	2.0 × 10 ^8^	3.4	0.6^a^	1.6 × 10 ^8^
CQI (2)	06/05/2017	4.4 × 10 ^3^	1.1 × 10 ^−2^	3.0 × 10 ^5^	1.4 × 10 ^10^	45	19.2	1.1 × 10 ^10^
CQI (3)	16/05/2017	7.6 × 10 ^3^	8.3 × 10 ^−4^	7.0 × 10 ^3^	5.6 × 10 ^8^	2.4	2.4	4.5 × 10 ^8^
CQI (4)	23/05/2017	2.2 × 10 ^4^	4.6 × 10 ^−2^	6.0 × 10 ^6^	1.8 × 10 ^11^	78	45.6	1.5 × 10 ^11^
CC (4)	23/05/2017	1.8 × 10 ^3^	5.2 × 10 ^−1^	9.0 × 10 ^6^	2.1 × 10 ^11^	78	45.6	6.2 × 10 ^10^
DZ (3)^b^	16/05/2017	8.9 × 10 ^3^	NA	NA	NA	2.4	2.4	NA
DZ (4)	23/05/2017	1.5 × 10 ^5^	1.3 × 10 ^0^	1.9 × 10 ^9^	2.1 × 10 ^13^	78	45.6	1.9 × 10 ^12^
HZ (3)	16/05/2017	3.5 × 10 ^1^	7.0 × 10 ^−1^	2.0 × 10 ^5^	3.5 × 10 ^10^	2.4	2.4	4.8 × 10 ^8^
HZ (5)	11/06/2017	5.0 × 10 ^3^	3.0 × 10 ^0^	1.3 × 10 ^8^	1.0 × 10 ^13^	102	58.2	1.4 × 10 ^11^

a This Event is minor and preceded by larger Events in the preceding days (see Fig. 2).

b No discharge at DZ for this Event.

### 3.1 Long term profiles of *E. coli* concentration and discharge over successive events

Four events were sampled at CQI between 19th April and 27th June 2017 (Fig. 2 a), capturing two major (>10 mm d^−1^) rainfall events at the commencement of the wet season. A simultaneous rise of *E. coli* concentration and Q was evident during Events 2 and 4, while there was minimal Q response for Events 1 and 3 but a distinct increase in *E. coli* concentration. The range of *E. coli* concentration increase was 0.6–2.4 orders of magnitude for all four events (increase of 8.0 x 10^2^–2.0 × 10^4^ CFU 100 mL^−1^).

**Fig. 2:**
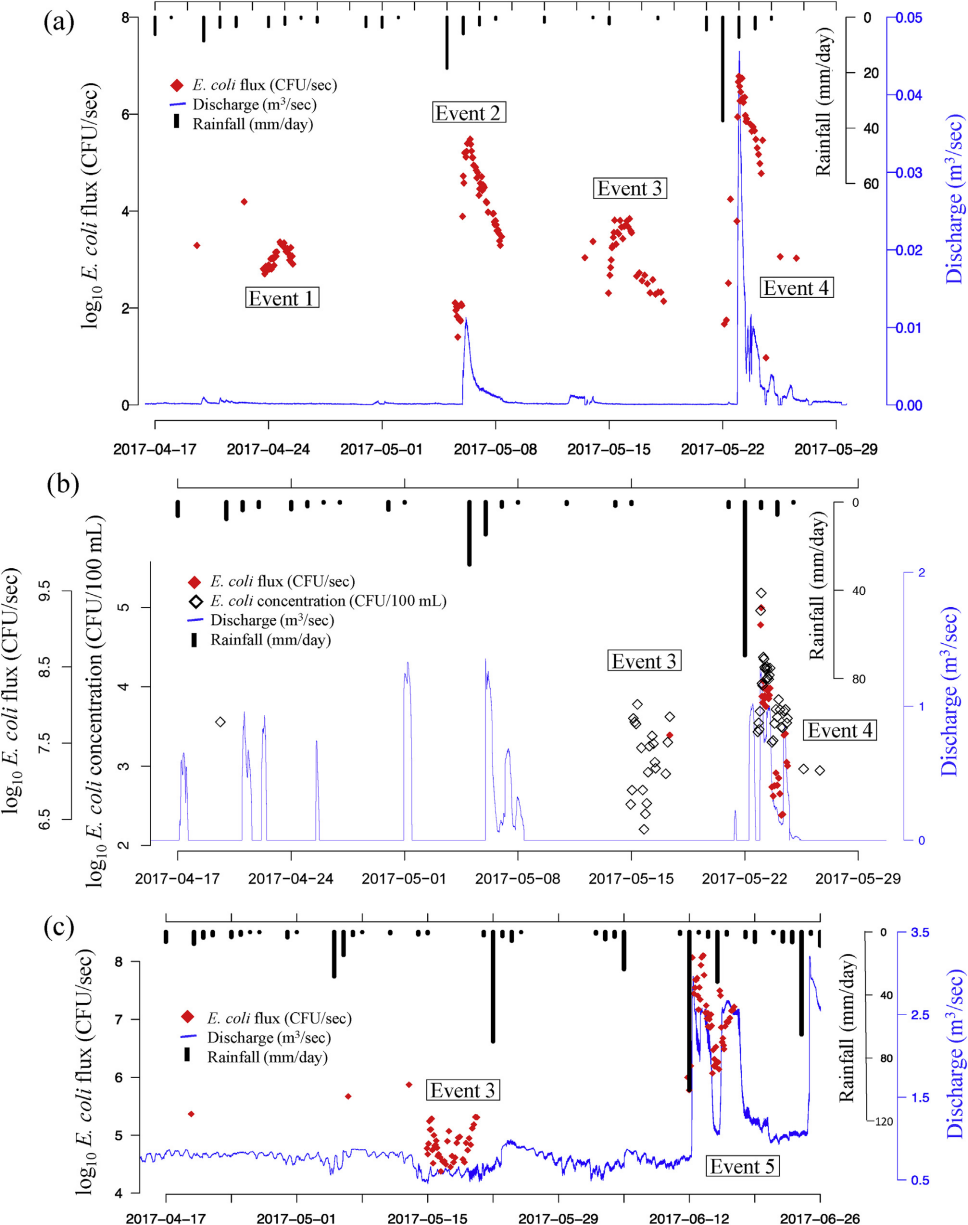
**(a)** Discharge and log_10_*E. coli* flux from CQI groundwater outlet over the period 17/4/2017–29/5/2017; **(b)** Discharge and *E.coli* concentration and flux data from DZ river over the period 17/4/2017–29/05/2017; **(c)** Discharge and *E. coli* flux data from HZ groundwater outlet over the period 01/4/2017–01/07/2017.

Events 3 and 4 (2.4 and 78 mm total rainfall) were sampled at DZ (Fig. 2 b). No increase in water level (and therefore Q) was recorded for Event 3 but the concentration of *E. coli* increased by almost 2 orders of magnitude. However, a similar concentration to the maximum recorded after Event 3 was measured in a grab sample on the 20/4 and in the samples taken a few hours prior to Event 4.

Rainfall events 3 and 5 (2.4 mm and 102 mm total rainfall) were monitored at HZ groundwater outlet (Fig. 2 c). An increase in Q occurred for Event 3, but increases and decreases in Q of a similar order of magnitude occurred during dry periods. *E. coli* concentrations remained low for Event 3 (pre-event: 1.2 × 10^1^ CFU 100 mL^−1^, event maximum: 3.4 × 10^1^ CFU 100 mL^−1^), but an increase in flux of 0.5 orders of magnitude was observed, translating to an increase in export of 1.3 x 10^5^ CFU s^−1^. Pre-event *E. coli* concentration for Event 5 was also low (1.2 × 10^1^ CFU 100 mL^−1^) but increased 1.6 orders of magnitude from pre-event to peak event *E. coli* concentration. An increase in flux of two orders of magnitude was observed, equivalent to an increase in export of 1.3 x 10^8^ CFU s^−1^. Additional grab samples taken in April, the day after two small-moderate rainfall events (20 mm and 2.0 mm), had comparable *E. coli* concentrations than those taken during Event 3.

### 3.2 Comparison: events monitored simultaneously at multiple sites

**Fig. 3:**
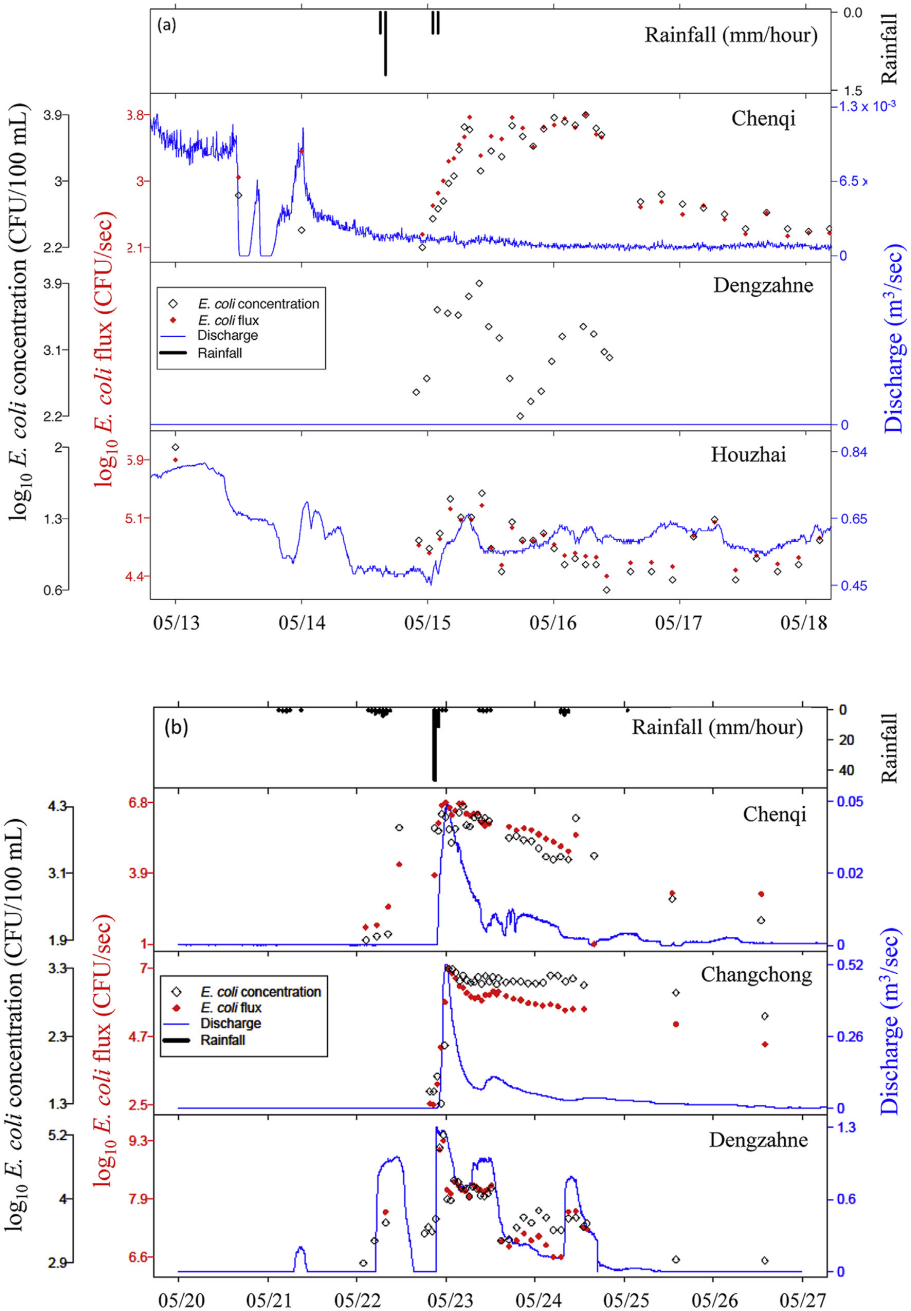
Comparison of discharge, *E. coli* concentration, and *E.coli* flux data from **(a)** CQI, DZ, and HZ over Event 3 (2.4 mm), and **(b)** CQI, DZ, and CC over Event 4 (78 mm). Note difference in scales for *E. coli* and discharge across sites.

Rainfall Event 3 was monitored simultaneously at CQI, DZ, and HZ (Fig. 3a), while Event 4 was monitored simultaneously at CQI, CC, and DZ (Fig. 3b). No response in Q was seen for Event 3 at CQI or DZ, while almost a 50% increase was observed at HZ. An increase in *E. coli* concentration was observed at all three sites following rainfall, and an increase in flux at CQI and HZ (but not DZ, as Q was zero). Immediately prior to the event, Q at HZ had receded and stabilised at ∼60% of the relatively constant level observed over the previous month. Within 2 h, *E. coli* concentration increased by an order of magnitude at CQI, and doubled at HZ. Discharge at both CQI and HZ fluctuated significantly before the event. *E. coli* concentration in a grab sample taken at HZ two days prior was higher (but the same order of magnitude) as event samples, and grab samples taken at CQI in the two days prior were also the same order of magnitude as pre-event samples.

For Event 4, CQI and CC followed similar Q responses, primarily responding to the main rainfall event, while DZ exhibited additional Q peaks both before and after the main event, responding to the minor rainfall events in the 24 h prior to and following the main event. Maximum Q at CQI was lower (4.6 × 10^−2^ m^3^ s^−1^) than CC (5.2 × 10^−1^ m^3^ s^−1^), but peak *E. coli* concentration was an order of magnitude higher (2.2 x 10^4^ and 1.8 × 10^3^ CFU 100 mL^−1^, respectively), resulting in comparable maximum flux (6.0 × 10^6^ and 9.0 × 10^6^ CFU s^−1^) and export (1.8 × 10^11^ and 2.1 × 10^11^ CFU) during the monitoring period. The recession of *E. coli* concentration and flux was faster at CQI. After nearly 4 days (88 h) *E. coli* concentration at CQI dropped by 2 orders of magnitude (to < 1% of peak concentration), while *E. coli* concentration at CC dropped less than one order of magnitude, remaining an order of magnitude higher than pre-event concentration at ∼20% of peak concentration. DZ exhibited a peak *E. coli* concentration almost 2 orders of magnitude higher than CC and one order higher than CQI. The maximum *E. coli* flux at DZ was 2–2.5 orders of magnitude higher, as peak Q was 2.0 times that at CC, and 2.6 times that at CQI. However, discharge returned to zero more rapidly at DZ than CC and after 88 h *E. coli* concentration was the same order of magnitude (within 10%) of pre-event levels at DZ.

### 3.3 Linear regression modelling

Table 3 summarises simple correlation analysis, which provides support for the linear regression analysis.

**Table 3:** Correlations between *E. coli*, turbidity, and discharge.

Site	Date	Rainfall amount (mm)	*E. coli* flux-discharge	*E. coli* conc-discharge	*E. coli* flux -Turbidity	*E. coli* conc-turbidity
CQI	24/04	3.4	0.06^NS^	−0.15^NS^	0.45**	0.4**
CQI	06/05	45	0.97***	0.83***	No turbidity	
CQI	16/05	2.4	0.18^NS^	−0.02^NS^	−0.13^NA^	−0.19^NS^
CQI	23/05	78	0.94***	0.59***	0.88***	0.77***
CC	23/05	78	0.85***	0.45**	No turbidity	No turbidity
DZ	16/05	2.4	No discharge		No turbidity	No turbidity
DZ	23/05	78	0.91	0.78	No turbidity	No turbidity
HZ	16/05	2.4	0.15	0.05	−0.3	−0.31
HZ	11/06	102	0.55	0.39	0.68	0.62
CQI Events combined			0.85	0.52	0.6	0.51
All Events combined			0.88	−0.07	0.7	−0.32

Significance of parameters is indicated in superscript (NS = not significant, * = p < 0.05, ** = p < 0.01, *** = p < 0.001).

#### 3.3.1 *E. coli* concentration and discharge

**Fig. 4:**
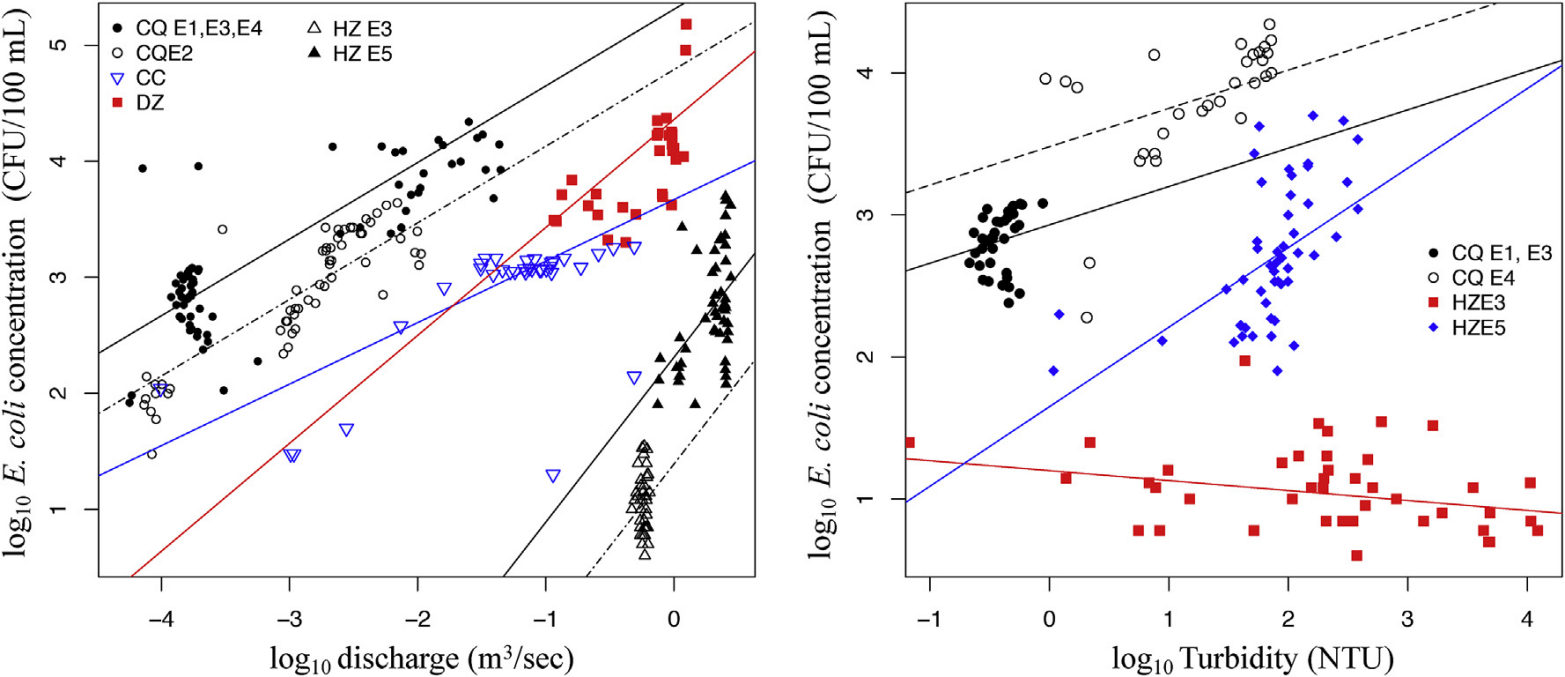
(left) *E. coli* concentration vs discharge, with best fit models (supplementary information, Table 7), and (right) *E. coli* concentration vs turbidity, with best fit models (supplementary information, Tables 8 and 9). All parameters log_10_. E1, E2, E3, E4, E5: Events 1,2, 3, 4, and 5 respectively.

Linear regression of log-log transformed *E. coli* concentration and Q identified that a single model was inadequate for all site data combined (R^2^ = 0.01, *p* > 0.05, AIC: 819). The best performing models (models 1, 2, and 3, Table 4) all incorporated both site and event as predictors. Allowing the slope to vary by site, event, or both produced similar model performance, as was the case for intercepts. Removal of ‘site’ as a predictor produced the largest increase in *delta* AIC (158.2), followed by 82.7 for the removal of ‘event’. The parameters for the model with the highest number of significant predictors, (model 3), are given in supplementary information (Table 7) and displayed in Fig. 4 with the data. Using model 3, also considered to be the most conceptually plausible, *E. coli* concentration at low discharge (represented by the model intercepts) was significantly different at all sites (*p* < 0.001) (Supplementary Information, Table 7). The rate of increase of *E. coli* concentration with Q was higher at HZ than at the other sites, which were not significantly different (Fig. 4), and the *E. coli* concentration following Events 2 and 5 (both high intensity and total rainfall amount), was significantly higher than during other events at the sites where they were sampled (CQI and HZ, respectively).

**Table 4:** Models of *E. coli* – discharge relationship ranked by AIC value. The *Slope* and *Intercept* columns indicate whether the slope and intercept of the model were allowed to vary by site, event, both, or neither. Models 1, 2, and 3 produced a similarly good fit to the data and were hence considered as candidate models.

Model	Slope	Intercept	AIC (*delta* AIC)	AIC	R^2^/conditional R^2^	*F-*statistic
1	Event	Site	227.2 (0)	227.2	0.86/0.86	155.4*** on 12 and 299 DF
2	Event and Site	Event and Site	229.3 (2.1)	229.3	0.86/0.86	124.9*** on 15 and 296 DF
3	Site	Event	232.8 (5.6)	232.8	0.86/0.85	165.4*** on 11 and 300 DF
4		Site, Event	237.6 (10.4)	237.6	0.85/0.85	220.5** on 8 and 303 DF
5	Site		309.9 (82.7)	309.9	0.81/0.81	190.1*** on 7 and 304 DF
6	Event		385.4 (158.2)	385.4	0.77/0.76	109.9*** on 9 and 302 DF

#### 3.3.2 *E. coli* concentration and turbidity

The relationship between *E. coli* concentration and turbidity was dependent on event characteristics as well as site characteristics. A stronger correlation existed at both CQI and HZ for events of higher rainfall intensity and amount (Table 3). Linear regression of log-log transformed *E. coli* concentration and turbidity showed that significantly different relationships exist at CQI and HZ regardless of event, and that significantly different relationships exist between events at each site (supplementary information, Tables 8 and 9). At HZ, *E. coli* concentration increased significantly with increasing turbidity for Event 5, while there was no significant change in the relationship for Event 3. At CQI, the rate of change was not significantly different between events, but the intercept and maximum turbidity and *E. coli* concentration were both higher for Event 4 which also showed a stronger correlation (Table 3). Assessment of the site-dependency of the relationships is limited by the number of sites for which turbidity data is available (two), but the available data suggests a higher concentration per unit turbidity at CQI than at HZ.

### 3.4 Rainfall-runoff event typologies from hysteresis, rising-falling limb characteristics, and flow duration curves

**Fig. 5:**
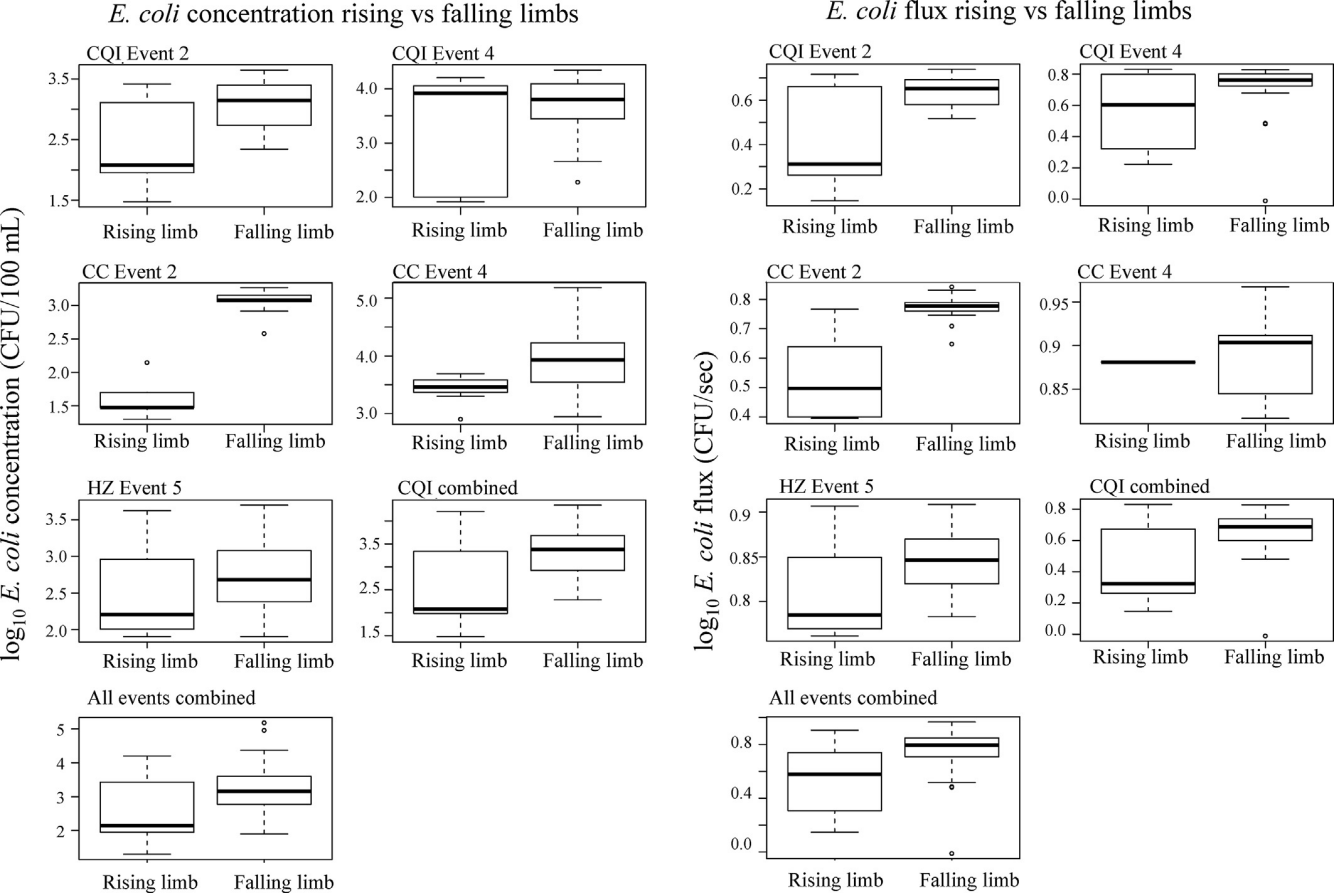
Distribution of (a) *E. coli* concentration and (b) *E. coli* flux values split into rising and falling limbs. Event 1 at CQI and Event 3 at HZ and DZ are excluded as they could not be categorised into a clear rising and falling limb. The Events for which the values are significantly different on the rising limb to the falling limb are marked (for *E. coli* concentration, CQI Event 2, CC Event 4, for *E. coli* flux, HZ Event 5).

**Fig. 6:**
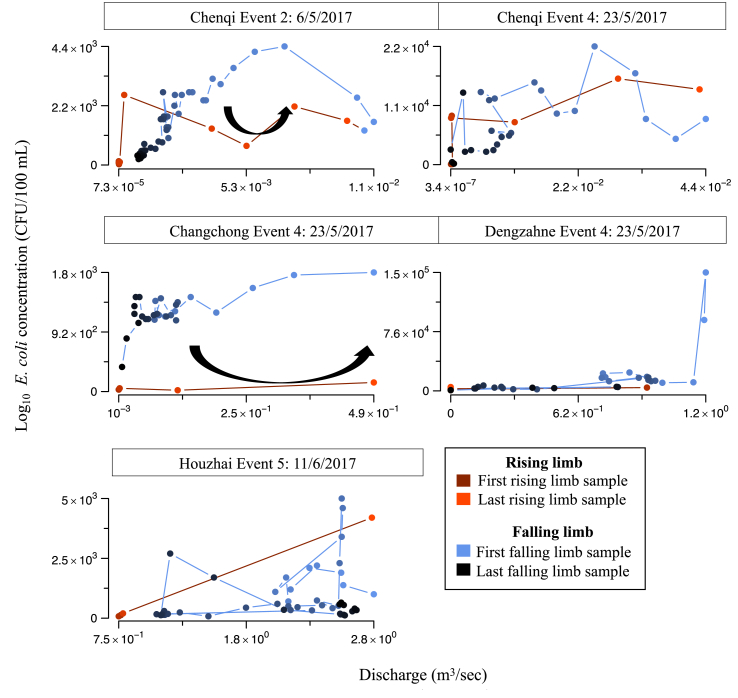
*E. coli* concentration-discharge hysteresis for Event-site combinations that could be split into rising and falling limbs. Arrows indicate the hysteresis direction for events where there is strong clockwise or anti-clockwise hysteresis.

Five of the rainfall event-site combinations resulted in a defined Q peak and therefore a well-defined hydrograph comprised of a rising and falling limb. The mean *E. coli* concentration was significantly higher on the falling limb rather than the rising limb for Event 2 at CQI and Event 4 at CC, suggesting a delay in peak *E. coli* relative to peak Q, and *E. coli* flux was similarly higher on the falling limb for one of these events (Fig. 5), otherwise no significant differences in concentrations were observed. There were insufficient samples for Event 4 at Dengzhanhe on the rising limb to test for significant difference with the falling limb. Highly variable *E. coli -* Q hysteresis patterns were observed across the range of event intensities and sample site locations (Fig. 6). Event 2 at CQI and Event 4 at CC (45 and 78 mm) demonstrated clear anti-clockwise hysteresis, while the remaining events cannot be clearly categorised as clockwise or anti-clockwise. Event 4 at CQI exhibits weak anti-clockwise and clockwise sections in the hystersis curve. Event 5 at HZ is complex: it could be categorised as clockwise but comprises only a few samples on the rising limb and the superposition of a second Q peak associated with a second smaller rainfall event.

**Fig. 7:**
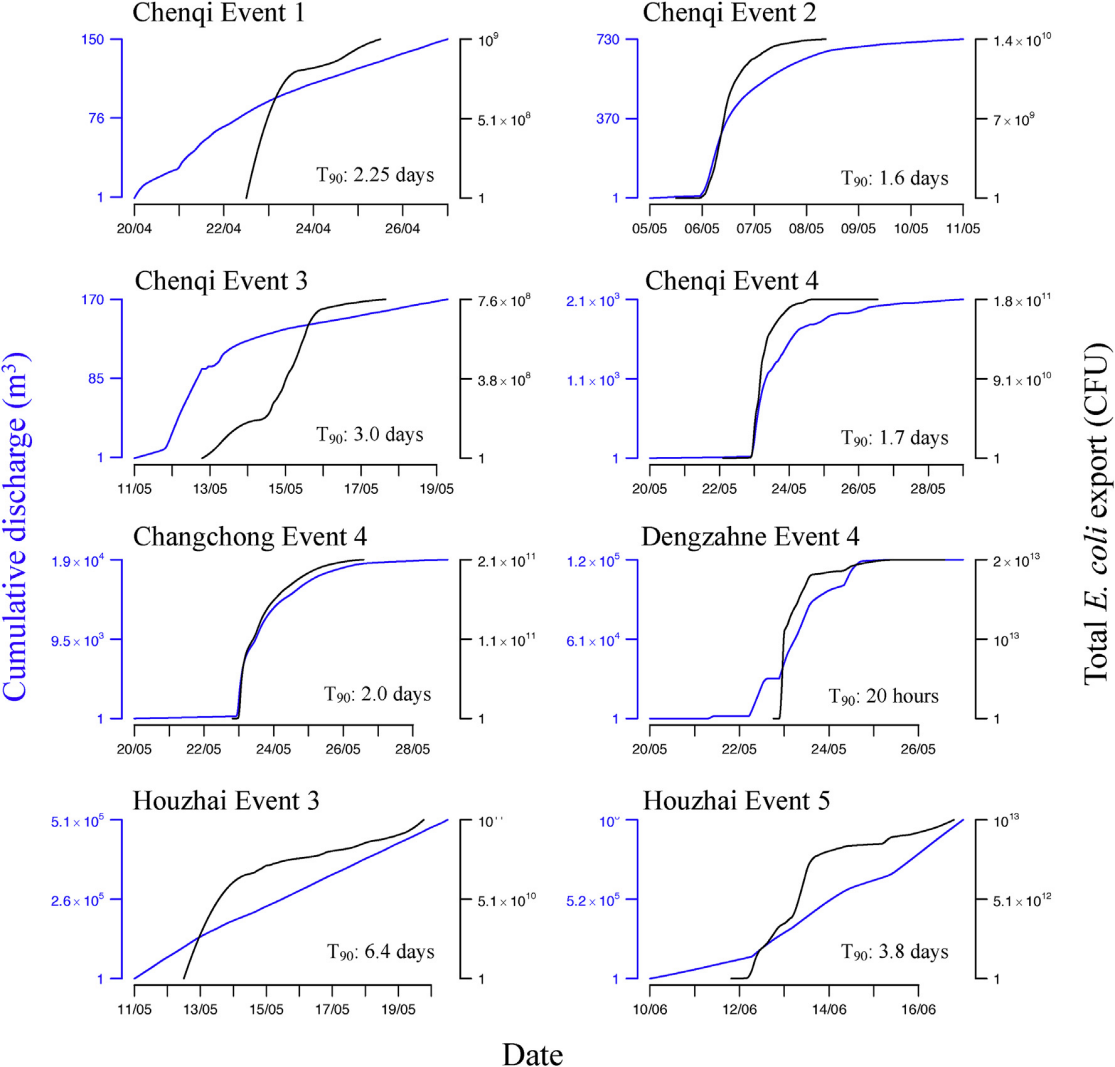
Cumulative discharge curves showing relationship between total discharge and *E. coli* export as events proceed.

The cumulative Q and *E. coli* export curves (Fig. 7) illustrate the short time period over which most event-associated *E. coli* export occurs. For the high intensity events 2 and 4 at CQI, CC and DZ, 90% of *E. coli* export occurred within 48 h, and within 24 h at DZ (T_90_ in Fig. 7). For the lower intensity events (1 and 3), and Event 5 at HZ, the time for 90% export was >3 days. For events with a distinctive Q peak (2, 4, and 5), cumulative Q began to diminish before cumulative *E. coli* export, but both discharge and *E. coli* concentration show similar asymptotic behaviour 24–48 h after the event. Event 3 (low rainfall intensity and amount) showed a linear increase in cumulative Q at both CQI and HZ, but CQI showed an increase in *E. coli* export while export at HZ remained linear. Cumulative Q and *E. coli* export followed a different evolution to other events and sites at HZ for Event 5, partially as a result of two distinct discharge peaks due to rain the day after the main event.

## 4 Discussion

High resolution characterisation of in-stream *E. coli* concentration in response to storm events is challenging; this study addresses this challenge and reports on a series of novel datasets of *E. coli -*Q relationships observed across a karst catchment over successive rainfall events. Events ranging from <5.0 mm to >100 mm rainfall over the study period were found to cause microbial water quality impairment at a range of spatial scales for 1–4 days following rainfall in mixed land-use paddy farming karst regions.

### 4.1 Interactions and relative importance of event characteristics, antecedent conditions, and source availability

Understanding of diffuse pollution has highlighted the importance of critical source areas (CSAs), defined as zones in the landscape where high sources of pollutants coincide with high potential for hydrological transfer (Heathwaite et al., 2005). The *E. coli* concentration and Q dynamics at the outlet of CQI indicate *E. coli* export from agricultural catchments following rainfall is strongly moderated by source availability and hydrological connectivity of sources to receiving waters, indicative of CSA influences and consistent with nitrate contamination patterns in this catchment (Yue et al., 2019). The increase in *E. coli* concentration was comparable for Events 1, 2, and 3 (spanning late April to late May) despite 1–2 orders of magnitude difference in rainfall and Q. This suggests that the timing of agricultural activities combined with seasonal rainfall patterns are likely to be the key controls on source availability and hydrological connectivity, respectively.

April represents a transitional period from the dry to the wet season in SW China, and also the period in the agricultural calendar where rapeseed is harvested and fields are tilled and fertilised with livestock manure for paddy crops (Yue et al., 2018). Therefore, relatively low rainfall may cause potentially large sources of FIOs, e.g. manure heaps prepared for land application, to become hydrologically connected to the groundwater system, via transfer from fresh manure heaps to sink holes or irrigation ditches. Evidence of increased FIO loading of watercourses in mixed-land-use or agricultural catchments during growing seasons and associated organic fertiliser application further supports this (Sinclair et al., 2009; Thilakarathne et al., 2018). The presence of impervious surfaces (e.g. roads) and nearby ditches can enhance connectivity between agricultural contaminant sources (such as FIOs and nutrients) and receiving waters in agricultural areas (Buchanan et al., 2012; Murphy et al., 2015). Furthermore, bare rock in karst areas is known to have a high runoff ratio (Li et al., 2011), which is particularly relevant to this region where karst rocky desertification results in extensive bare rock exposure and thus increased likelihood of overland flow pathways connecting FIO and sediment sources to streams and sinkholes (Dai et al., 2017). For local residents dependent on catchment water supplies, the commencement of the monsoon and paddy seasons presents a period of increased risk where particular care should be taken to treat water, or use an alternative supply, for 2–3 days following rainfall. Measures to reduce the potential for hydrological transfer from sources, such as careful placement and containment of manure heaps, may also reduce risk.

The anti-clockwise hysteresis observed for Event 2 (45 mm) at CQI and mixture of weak anti-clockwise and clockwise hysteresis for Event 4 (78 mm) suggest proximal sources potentially contribute more to export for Event 4, which resulted in an order of magnitude higher concentration, flux, and total export than Event 2. A threshold of 80 mm for activation of overland flow has been suggested in this karst area (Zhang et al., 2011), consistent with the magnitude of Event 4. Thus, activation of additional hydrological pathways, such as overland flow, could further contribute to the delivery of nearby FIO sources to receiving waters during high intensity rainfall (Lloyd et al., 2016).

Antecedent catchment conditions can influence *E. coli* transfer to receiving waters by increasing or decreasing the activation of overland and subsurface flow mechanisms (Hathaway et al., 2010). The timing of successive events in our study allows for the role of different antecedent conditions to be considered, although there are too few events across a suite of antecedent conditions and sites to enable analysis beyond preliminary empirical observation. Events were monitored both during the onset of the wet season (dry antecedent conditions, e.g. Event 1) and after numerous events had occurred (e.g. Event 4), when soil moisture is likely to have increased (Zhang et al., 2011). Low soil moisture due to dry antecedent conditions has been found to result in higher surface runoff generation and suspended sediment transport due to infiltration-excess overland flow (McDowell and Sharpley, 2002; Puntenney et al., 2016). The moderate but significant correlation between *E. coli* concentration and turbidity for Event 1 would be consistent with this process. Combined with the availability of sources at this time, rapidly-induced overland flow could be a further explanatory factor behind the high *E. coli* concentration observed following events early in the season. Increased soil moisture prior to rainfall is generally associated with a more rapid generation of saturation-excess overland flow and increased river discharge for a given rainfall amount relative to dry conditions (Penna et al., 2011). In this karst region, increased soil moisture has been found to decrease the amount of rainfall required before preferential subsurface flow along the soil-epikarst boundary is generated (Fu et al., 2015). The groundwater level in this region also increases during the wet season (Yue et al., 2015), potentially increasing activation of subsurface flow with successive rainfall events. This is a process that has been observed to cause increased nitrate transfer to agricultural streams (Outram et al., 2016), and could contribute to the elevated *E. coli* concentrations recorded later in the wet season. To attain an in-depth, process-scale understanding of how rainfall events of varying magnitude impact *E. coli* transfer in catchments, a combination of approaches would be required; controlled hillslope-scale studies quantifying the flux of FIOs through overland and subsurface pathways under varying land-use scenarios, quantification of sediment-driven remobilisation during events, and high resolution monitoring at the catchment outlet scale.

### 4.2 The role of catchment size, stream order/flow path length, variation in hydrological properties, and land-use distribution in moderating the *E. coli* – discharge response at catchment outlets

The response of *E. coli* and Q to rainfall was more rapid in the headwater catchments, and there appears to be minimal Q response at the basin outlet scale (HZ spring) for events less than ∼ 20–40 mm. Catchment-scale transport, and residence time of water in catchments can be determined primarily by flow path gradient and length (McGuire et al., 2005). In karst terrain, there is also inherent uncertainty in the delineation and characterisation of catchment boundaries and aquifers due to their spatial heterogeneity and leakiness, introducing a degree of uncertainty in attribution to spatial characteristics such as catchment size, or source distribution (Adinehvand et al., 2017). Both the surface and groundwater systems, that contribute to the outlet at HZ, flow through the flat agricultural land in the Dolomitic unit (T2g3) (Fig. 1 d), and can result in long residence times (est. average of 493 days) for some hydrological pathways (Zhang et al., 2019). HZ has 37% of land area with ≤ 5º slope, compared with 12%, 2%, and 21% for CQI, CC, and DZ respectively. In addition to the lower gradient in the lower reaches of the catchment, hydrological properties change as a result of differences in lithology. A highly-developed fissure network and clay cover in the Western plains where the dolomite unit (T2g3) outcrops prevents rapid infiltration, while the interbedded limestone-shale units in the headwaters show peak-cluster karst landforms with poorly developed fissure networks, and rapid infiltration of overland flow generated during rainfall through karstic features such as vertical pipes and sinkholes (Zhang et al., 2016). Thus during low flow conditions or for small rainfall events, transport of *E. coli* to the outlets of headwater catchments may still be significant, contributing to the higher concentrations at low discharge for the headwater catchments. Spatial heterogeneity in rainfall could also be a contributing factor to differences in discharge responses and *E. coli* emergence patterns observed between sub-catchment outlets; ideally each sampling location would be instrumented with a rain gauge to assess this.

Deposition with sediments, storage in the fissure network, or die-off in the water column is likely to prevent *E. coli* reaching HZ from long flow paths due to the slow flow velocity and well-developed fissure network (Liu et al., 2010). During high flow conditions Q may be sufficient for *E. coli* flushed into the hydrological system from surface sources to remain suspended along long flow-paths. For smaller events (e.g. Event 3: <5.0 mm), the lack of response in *E. coli* concentration with increasing scale above the headwater catchments could be due to a lack of proximal, hydrologically connected sources, but also attenuation of *E. coli* by processes such as attachment to sediment particles and settling in stream bed sediments as the hydraulic gradient, and hence stream flow velocity, decreases (Schiperski et al., 2016; Wyness et al., 2018).

The higher peak *E. coli* concentration and faster recession in *E. coli* flux observed at CQI compared with CC indicates greater availability and connectivity of proximal sources at CQI compared with CC. This is supported by the strong anti-clockwise hysteresis observed at CC and lack of discernible hysteresis at CQI, as well as the distribution of *E. coli* concentration and flux values on the rising and falling limbs for CC. Catchment shape, distribution of potential sources, and hydrology are suspected to be important. CQI is smaller than CC, but the catchment outlet is located at the centre of the continuously-cultivated valley depression, and the majority of paddy and agricultural land is located within 1 km of the catchment outlet, with no breaks in cultivated land. This distribution of paddy land, primarily in the discharge area of CQI, can influence pCO_2_ and other hydrogeochemical properties of water at the outlet (Zhao et al., 2010), which indicates its connectivity and influence on downstream water quality. The majority of agricultural land in CC, in contrast, is located >1.5 km from the catchment outlet, connected to the outlet via an underground conduit and surface flow during high flow conditions.

In CC catchment, several villages in the headwaters (∼3 km from the outlet) contain point sources. The slower recession of *E. coli* concentration and consequently *E. coli* flux at CC compared to CQI, despite similar recession in discharge, could be a result of lack of source depletion due to *E. coli* persistence, combined with the underground conduit system providing high connectivity between the villages and the sampling location. The maximum *E. coli* concentration at DZ was one order of magnitude higher than that at CQI, comparable to the increase in catchment area. However, the maximum *E. coli* flux, corresponding with timing of peak discharge, was three orders of magnitude higher, and occurred within an hour of the commencement of rainfall. Given the urban character of the DZ sampling site this demonstrates the potential potency of urban point sources. The presence and proportion of urban land have been found to represent important causes of elevated FIO concentrations in many mixed land-use catchment studies (Kay et al., 2005; McGrane et al., 2014; Neill et al., 2018; Paule-Mercado et al., 2016). The average temperature at DZ sampling location was also significantly higher than at CQI or CC during the sampling months (19.8 compared with 16.6 for both CQI and CC). Although increasing temperature is typically associated with increased mortality of *E. coli,* higher water temperatures have also been associated with higher *E. coli* loads in catchments, attributed to more favourable conditions for regrowth (Badgley et al., 2019; Chen and Chang, 2014). The flashy discharge response at DZ, and shorter time for 90% of total *E. coli* export to have occurred relative to the CQI and CC tributaries (19 h versus ∼2 days), also suggests rapid input from proximal sources, probably resulting from the dominance of overland flow transport pathways due to impervious surfaces in the urban surrounds (Du et al., 2015). The dominantly impervious concrete drains that modify the surface water system delivering water from feeder tributaries, such as CQI, are also likely to exacerbate the flashy nature of the Q response, though this requires further research.

### 4.3 Mechanisms of *E. coli* transport: inferences from over-arching relationships between *E. coli* and Q, and *E. coli* and turbidity

Collectively, the results suggest *E. coli* transport is more strongly associated with discharge than sediment (assuming turbidity as a proxy for suspended sediment), but that sediment-associated transport is more significant for high intensity rainfall, high discharge events. The trend in the rate of increase of *E. coli* concentration with increasing discharge for the preferred model (model 3) follows catchment-scale trends in flow-path gradient and hydrological properties. However, the presence of ‘Site’ and ‘Event’ as predictors in all candidate models for the relationship between *E. coli* and discharge, and their similar performance in terms of model fit, illustrates that further event data at a range of catchment scales is needed to elucidate with greater confidence how event characteristics interact with catchment hydrology (and source availability) to influence site-specific *E. coli-*discharge dynamics. Ideally, sufficient events and sites would be sampled to include event characteristics (e.g. rainfall intensity and amount), and hydrological/catchment properties (e.g. contributing area, infiltration rates, flow path length) as continuous predictors in modelling.

The event dependency of the association between *E. coli* concentration and turbidity, with the strength of correlation increasing with rainfall amount and discharge is consistent with other studies in karst, where *E. coli* has often correlated with suspended sediment concentration under high discharge conditions (Mahler et al., 2000; Pronk et al., 2006). The activation of overland flow during higher intensity events may contribute to the higher sediment loads observed during events, and is likely to be more significant than in non-karst catchments (Buckerfield et al., 2019). The resuspension of streambed, fissure, or conduit sediment stores may also contribute to the increasing strength of correlation with increasing discharge - indeed, the potential for survival of *E. coli* in karst conduits for several months has been demonstrated (Bradshaw et al., 2016; Thilakarathne et al., 2018).

## 5 Conclusions

The Houzhai catchment represents a typical landscape in the southwest China karst region. This study identified rainfall characteristics, land-use, and karstic hydrology to be fundamental controls on *E. coli*-Q dynamics and export in mixed land-use karst catchments typical of this terrain. Comparison of *E. coli* – Q dynamics at different catchment outlets showed that both urban (e.g. in DZ) and agricultural land (e.g. in CQI) can contribute high *E. coli* loadings to receiving waters, but that the distribution of these land-use categories and associated faecal sources relative to hydrological pathways is equally as important in determining maximum *E. coli* concentration and subsequent recession rates, as has been observed elsewhere (Neill et al., 2018). The portion of the population relying on catchment water resources as a drinking water supply and for domestic use are at higher risk, and represent a significant proportion of the rural population in southwest China. There is a heightened risk of exposure to faecally-contaminated water for 2–3 days after rainfall events, particularly during key periods in the agricultural calendar when high source availability and connectivity to receiving waters coincide. In urban areas, investment in waste containment and treatment infrastructure is necessary, but in agricultural areas, microbial water quality could be improved by identifying, managing, and raising awareness of critical source areas of FIOs. To do this effectively will first require a better understanding of the uncertainties associated with complex hydrogeological pathways and the spatial and temporal trends in FIO burden delivered to land from agricultural practices that occur across headwater to catchment scales.

## Declaration of competing interest

The authors declare that they have no known competing financial interests or personal relationships that could have appeared to influence the work reported in this paper.
