# Supplementary material for: Rainfall-driven E. coli transfer to the stream-conduit network observed through increasing spatial scales in mixed land-use paddy farming karst terrain
Source: Water Res X. 2019 Oct 10;5:100038. doi: 10.1016/j.wroa.2019.100038 (PMC6807365; doi:10.1016/j.wroa.2019.100038)
Supplement: Multimedia component 1 [file mmc1.docx]

# Supplementary Information

Table 5: Explanation of the derivation of Event summary parameters and sources of datasets.

| **Parameter** | **Explanation of derivation** |
| --- | --- |
| Date | Day during which the majority of rainfall occurred, if Event spans more than one day. |
| Maximum *E.coli* concentration | Maximum *E.coli* concentration measured in samples from the Event, in colony forming units per 100 mL (CFU 100 mL^-1^). Samples taken more than 12 hours before the Event were excluded. |
| Maximum discharge | Maximum discharge measured during the Event. Discharge is derived from water level measurements taken at 5 minute intervals. Discharge and water level data is courtesy of the hydrological modelling component of the karst critical zone project (Zhicai Zhang, by correspondence). |
| Maximum *E.coli* flux | Maximum over the duration of the Event of the product of *E.coli* concentration (converted to CFU m^-3^) and discharge (discharge value is derived from water level measured within 5 minutes of *E.coli* sample). |
| Total *E.coli* export | Discharge and *E.coli* concentration data were linearly interpolated and discretised into units of seconds. Total export was calculated by trapezoidal integration of the derived *E.coli* flux at one second intervals. The duration of the Event was taken as the duration of monitoring. |
| Rainfall amount | Total rainfall over the Event. Rainfall data from Lahoetain and CQI is available. There are significant differences in the rainfall amount recorded at these two stations for some Events. Data from CQI is used as this is closest to CQI, CC, and DZ. Lahoetain data is courtesy of the hydrological modelling component of the karst critical zone project (Zhicai Zhang, by correspondence), and rainfall data from CQI is courtesy of Puding Karst Ecosystem Research Station. |
| Maximum rainfall intensity | Maximum of rainfall discretised into hourly intervals (rainfall readings are taken every 5 minutes). |
| Event loading (CFU km^-2^) | Total *E. coli*  export for the Event divided by the catchment area (km^2^) |

*Table 6:* Average hourly water temperature at the four sampling sites between 4/4/2017 and 19/06/2017 for which there were complete observations at all four sites. *denotes the temperature was significantly different at this site to at all other sites. Note the dataset is limited by incompleteness of observations; when complete observations for CQI and CC only are compared, the difference in their means is significant.

| Site | Average water temperature (ºC) |
| --- | --- |
| CQI | 16.6 |
| CC | 16.6 |
| DZ | 19.8* |
| HZ | 18.3* |

*Table 7:* Model parameters (intercept*s* and slopes) for linear fixed effects model estimating the relationship between *E. coli* concentration and discharge. The overall intercept is one of the four site options (in this case, Changchong, CC), but which site this parameter corresponds to does not change the model output. The model then estimates whether the intercepts are significantly different for the other sites (CQ, DZ, HZ), for example the intercept from samples taken at HZ is 1.69 log units lower than at CC. The same procedure is followed for the slope calculation. In this case the slope is significantly different at HZ than the other sites (by 2.32 log units), and the intercept is significantly different for every site. Significance levels: *** Pr(>|t|) < 0.001, ** Pr(>|t|)<0.01.

| Model formula: log_10_ (*E. coli* concentration) = *m* x log_10_ (Q) * factor (Site) + factor(Event)  where m…..slope  Q…..discharge  The factor (Site) term allows slope and intercept to vary for each of the four sites (CQ, CC, DZ, HZ), and the factor (Event) term allows the intercept to vary between events. | | | |
| --- | --- | --- | --- |
| **Parameter** | **Estimate** | **Std. Error** | **t-value** |
| Overall intercept | 3.67 | 0.17 | 21.2*** |
| Slope | 0.53 | 0.09 | 5.5*** |
| Factor (Site) CQI | 1.64 | 0.20 | 8.7*** |
| Factor (Site) DZ | 0.69 | 0.16 | 4.4*** |
| Factor (Site) HZ | -2.29 | 0.20 | -11.7*** |
| Factor (Event) 2 | -0.52 | 0.08 | -6.2*** |
| Factor (Event) 3 | 0.00 | 0.08 | 0.05 |
| Factor (Event) 4 | -0.14 | 0.11 | -1.3 |
| Factor (Event) 5 | 0.92 | 0.18 | 5.0*** |
| Slope adjustment (CQ) | 0.13 | 0.11 | 1.2 |
| Slope adjustment (DZ) | 0.40 | 0.25 | 1.9 |
| Slope adjustment (HZ) | 0.89 | 0.18 | 2.9** |
| **Whole of model parameters** | | | |
| Multiple/Adjusted R-squared | 0.86/0.85 | | |
| F-statistic | 190.1 on 7 and 304 degrees of freedom  (p-value: <0.001) | | |

*Table 8:* Model parameters (intercept*s* and slopes) for linear fixed effects model estimating the relationship between *E. coli* concentration and turbidity at Chenqi (CQI)*.* The overall intercept is one of the three event options (in this case, Event 1), but which site this parameter corresponds to does not change the model output. The model then estimates whether the intercepts are significantly different for the other events (Event 3, Event 4) for example the intercept for Event 4 is 0.55 log units higher than Event 1. The same procedure is followed for the slope calculation, if the slope is allowed to vary. Significance levels: *** Pr(>|t|) < 0.001.

| Model formula: log_10_ (*E. coli* concentration) = *m* x log_10_ (T) + factor (Event)  where m…..slope  T…..turbidity  The factor (Event) term allows intercept to vary between events. | | | |
| --- | --- | --- | --- |
| **Parameter** | **Estimate** | **Std. Error** | **t-value** |
| Overall intercept | 2.93 | 0.04 | 67.7*** |
| Slope | 0.27 | 0.02 | 12.7*** |
| Factor (Event) 3 | 0.003 | 0.06 | 0.05 |
| Factor (Event) 4 | 0.55 | 0.07 | 8.3*** |
| **Whole of model parameters** | | | |
| Multiple/Adjusted R-squared | 0.79/0.78 | | |
| F-statistic | 123.4 on 3 and 99 degrees of freedom  (p-value: <0.001) | | |

*Table 9****:*** Model parameters (intercept*s* and slopes) for linear fixed effects model estimating the relationship between *E. coli* concentration and turbidity at Houzhai (HZ). The same principles apply as for parameters in *Table 5.* Significance levels Pr(>|t|): *** < 0.001; *<0.1

| Model formula: log_10_ (*E. coli* concentration) = *m* x log_10_ (T) * factor (Event)  where m…..slope  T…..turbidity  The *factor (Event) term allows intercept and slope to vary between events. | | | |
| --- | --- | --- | --- |
| **Parameter** | **Estimate** | **Std. Error** | **t-value** |
| Overall intercept | 1.2 | 0.12 | 10.0*** |
| Slope | -0.07 | 0.05 | -1.4 |
| Factor (Event) 5 | 0.45 | 0.23 | 1.9* |
| Slope adjustment (Event) 5 | 0.63 | 0.11 | 5.53*** |
| **Whole of model parameters** | | | |
| Multiple/Adjusted R-squared | 0.86/0.86 | | |
| F-statistic | 181.8 on 3 and 87 degrees of freedom  (p-value: <0.001) | | |
